# Painful red nodules in female patient with recent travel history: a case report

**DOI:** 10.4076/1757-1626-2-8248

**Published:** 2009-06-19

**Authors:** Craig Dean, William Thomas Crow

**Affiliations:** Department of Medical Education, Florida Hospital East Orlando7975 Lake Underhill Rd Suite 200, Orlando FL 32822USA

## Abstract

**Introduction:**

Dermatologic pathology can be both challenging and frustrating in the Family Practice setting.

**Case presentation:**

We report the case of a 38-year-old female that presented initially with a few, small red nodules on both the upper and lower extremities, which were painful to touch. This patient had an extremely vague picture, which included recent upper respiratory infection and recent travel to Europe. Erythema Nodosum was suspected and work-up initiated to determine underlying cause. ASO titer ultimately confirmed recent Streptococcal infection. Although the primary diagnosis was not made until follow-up visit, treatment was started based on understanding of common causes of Erythema Nodosum.

**Conclusion:**

Using the patient's history, a differential diagnosis knowing the common causes of EN can help direct diagnostic evaluation.

## Introduction

Erythema Nodosum (EN) was first described by English dermatologist Robert Willan in 1798, which was published in his work *Cutaneous Diseases*. EN is characterized by red or violet subcutaneous nodules that usually occur on the anterior portion of the lower extremities however can also be present on thighs, trunk, or upper extremities [1]. Most cases of EN usually have no identifiable cause however several reported cases stem from recent Streptococcal infection. We present a case of EN in which Streptococcal infection was suspected and treated while ruling out other systemic pathology.

## Case presentation

A 38-year-old Caucasian female presented with bilateral upper extremity and lower extremity lesions, which were painful to touch and joint pain in her knees, wrists, and ankles. Patient states that 2 weeks prior to initial visit she started with upper respiratory symptoms including sore throat, cough, congestion, and body aches. She states over the course of 2-3 days her signs and symptoms improved with over the counter medications and she went to Europe on vacation for 10 days. Towards the end of her trip, she started getting joint pain as described above. Two days prior to her clinic visit, she developed the first lesion on her right wrist. No significant past medical history was given. The patient states she was in relatively good health. She had had a pre-employment physical one month prior to her visit, which included TST which was negative. She states that when she was younger she had her tonsils and adenoids removed. Patient denied being on any recent antibiotics or birth control pills. Family history included hypothyroidism, which her mom has. Social history reported no history of tobacco use and social use of alcohol. At time of presentation, patient's vital signs were stable. Ear, nose, and throat exam revealed cobble stoning to the posterior pharynx. No cervical lymphadenopathy was appreciated. Dermatologic examination revealed multiple raised erythematous lesions on the palmer aspect of hands, the forearms, the anterior portion of lower extremities, and the dorsal aspect of the feet. Nodules were tender to palpation and there was decrease range of motion of the bilateral wrists and ankles secondary to pain. Muscle strength was 5/5 bilaterally and reflexes were + 2/4 in the upper extremities and lower extremities bilaterally.

**Figure 1.**
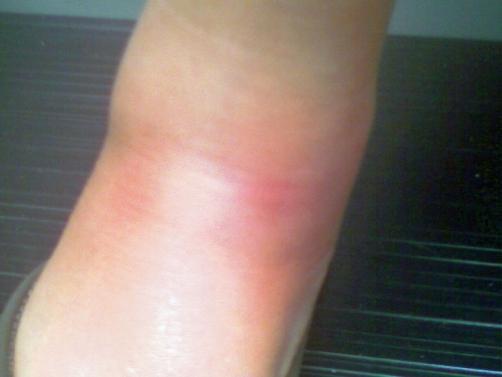
Erythematous nodule located on the medial aspect of the flexor surface of right ankle.

**Figure 2.**
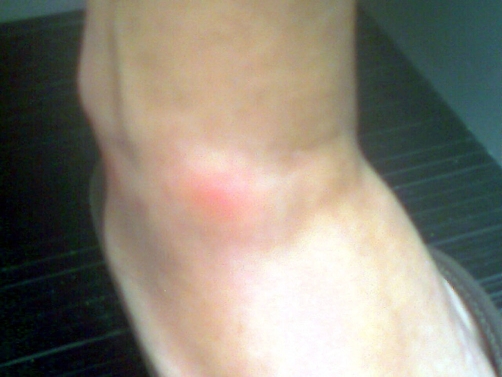
Erythematous nodule located on the medial aspect of the flexor surface of left ankle.

**Figure 3.**
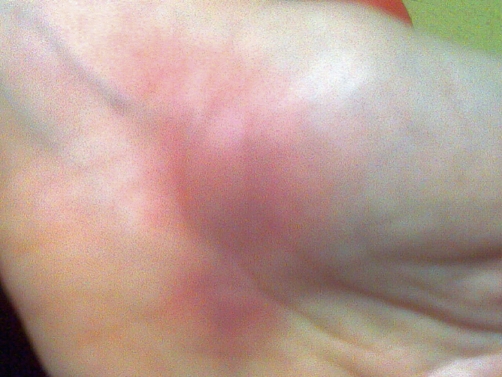
Erythematous nodule located on the palmer surface of left hand.

EN was suspected and patient was started on Amoxil 875 mg by mouth twice daily for 10 days along with 800 mg of Motrin every 8 hours for pain. CBC with differential, CMP, TSH, ASO titer, ESR, and CXR were ordered and patient was instructed to follow-up in 1 week. Upon patient follow-up, patient states she had been seen in the local emergency room 3 days prior to visit for worsening joint pain. She stated they had given her some pain medications through her IV and instructed her to follow up with her primary doctor. Labs reviewed with patient showed she had a significant elevation in her ESR at 73 and her ASO and anti-DNase B titers were elevated at 653 and 1710 respectively. CBC showed slight elevation in WBC count at 13.9 and her CMP and TSH were wnl. CXR reviewed showed no acute pulmonary process. Informed patient that inflammatory nodules were most likely result of recent Streptococcal infection and she is on appropriate antibiotics and nodules may take up to 6 to 8 weeks to resolve. Patient was prescribed Sterapred Ds 12 pack and was referred to local primary care doctor for follow up (Figure 1, Figure 2 and Figure 3).

## Discussion

Erythema Nodosum (EN) is the most common type of septal panniculitis [1]. EN has an annual incidence of 5/100,000 persons and is most prevalent in the second to fourth decades of life, with women more often affected than men [2]. In reviewing the literature, there are several causes of EN. The most common infectious etiology is streptococcal infection, with others including tuberculosis and pulmonary fungal infections including histoplasmosis and blastomycosis less common. Other etiologies include sarcoidosis, inflammatory bowel disease, and various drugs including sulfa medications and birth control pills [1,3]. However, the majority of cases have no identifiable cause. Recent case reports suggest that streptococcal infections and sarcoidosis are the leading identifiable causes of EN in the United States and Europe; however they reported that 55% or reported cases were of unknown etiology [4]. Interestingly, one case report found that most initial encounters of patients with EN were misdiagnosed as having cellulitis [5]. The disease course is usually self-limited and the nodules resolve within 6-8 weeks. The goal of treatment is focused on pain management, with high dose NSAIDs (Motrin 800 mg every 6-8 hrs) recommended. Pain refractory to NSAIDs may respond to narcotic analgesia or a short course of systemic steroids [6]. Other treatment modalities including potassium iodine and Vitamin B12 have been reported with varying results [7].

## Conclusion

Erythema Nodosum (EN) can be difficult to diagnosis in a Family Practice setting. EN should be considered in any patient with multiple erythematous cutaneous nodules, regardless of location. Using the patient's history, a differential diagnosis knowing the common causes of EN can help direct diagnostic evaluation. Reassurance to the patient that this disease is self-limiting and adequate control of pain with NSAIDs is often the only treatment necessary.

## Abbreviations

ASO: anti-streptolysin O
CBC: complete blood count
CMP: complete metabolic panel
ESR: erythrocyte sedimentation rate
IV: intravenous
NSAIDs: Non-steroidal anti-inflammatory drugs
TSH: thyroid stimulating hormone
TST: tuberculin skin test
